# A standardized approach to treat complex aortic valve endocarditis: a case series

**DOI:** 10.1186/s13019-018-0715-8

**Published:** 2018-04-19

**Authors:** Anna Gomes, Jayant S. Jainandunsing, Sander van Assen, Peter Paul van Geel, Bhanu Sinha, Sandro Gelsomino, Daniel M. Johnson, Ehsan Natour

**Affiliations:** 1 Department of Medical Microbiology, University of Groningen, University Medical Center Groningen, Groningen, Netherlands; 2 Department of Anesthesiology, University of Groningen, University Medical Center Groningen, Groningen, Netherlands; 3 Department of Internal Medicine, Infectious Diseases, Treant Care Group, Hoogeveen, Netherlands; 4 Department of Cardiology, University of Groningen, University Medical Center Groningen, Groningen, Netherlands; 5 Department of Thoracic Surgery, Maastricht University Medical Center, Maastricht, Netherlands; 6 Department of Cardio-Thoracic Surgery, University of Groningen, University Medical Center Groningen, Groningen, Netherlands

**Keywords:** Infective endocarditis, Stentless bioprostheses, Abscess, High-risk, Surgery

## Abstract

**Background:**

Surgical treatment of complicated aortic valve endocarditis often is challenging, even for experienced surgeons. We aim at demonstrating a standardized surgical approach by stentless bioprostheses for the treatment of aortic valve endocarditis complicated by paravalvular abscess formation.

**Methods:**

Sixteen patients presenting with aortic valve endocarditis (4 native and 12 prosthetic valves) and paravalvular abscess formation at various localizations and to different extents were treated by a standardized approach using stentless bioprostheses. The procedure consisted of thorough debridement, root replacement with reimplantation of the coronary arteries and correction of accompanying pathologies (aortoventricular and aortomitral dehiscence, septum derangements, Gerbode defect, total atrioventricular conduction block, mitral and tricuspid valve involvement).

**Results:**

All highly complex patients included (14 males and 2 females; median age 63 years [range 31–77]) could be successfully treated with stentless bioprostheses as aortic root replacement. Radical surgical debridement of infected tissue with anatomical recontruction was feasible. Although predicted operative mortality was high (median logarithmic EuroSCORE I of 40.7 [range 12.8–68.3]), in-hospital and 30-day mortality rates were favorable (18.8 and 12.5% respectively).

**Conclusions:**

Repair of active aortic valve endocarditis complicated by paravalvular abscess formation and destruction of the left ventricular outflow tract with stentless bioprosthesis is a valuable option for both native and prosthetic valves. It presents a standardized approach with a high success rate for complete debridement, is readily available, and yields comparable clinical outcomes to the historical gold standard, repair by homografts. Additionally, use of one type of prosthesis reduces logistical issues and purchasing costs.

## Background

Infective endocarditis causes in-hospital mortality of 20% and 40% after 1-year, rising further to 79% for aortic valve endocarditis [1, 2]. This high rate is largely due to extended local destruction of heart tissue, e.g. paravalvular abscess formation, with secondary heart failure. Risk factors for endocarditis include rheumatic, congenital, and degenerative valve lesions, intracardiac prosthetic material, intravenous drug use, and healthcare contact [3]. Diagnosis of endocarditis is based on the modified Duke criteria, bearing a sensitivity and specificity of 80% for the total patient population [4]. As this is not optimal, the expert opinion of a multidisciplinairy team is essential for diagnosis. Therapy of endocarditis relies on antimicrobial therapy and surgery for cardiac anatomical damage (vegetation, abscess, fistula, shrunken valve, valve tears or holes, prosthetic valve detachment), as well as uncontrolled infection. In this way, 25–50% of patients are operated upon in the acute phase of infection and an additional 20–40% later in the course due to haemodynamic complications [5].

Paravalvular abscess formation complicates aortic valve endocarditis. Early surgical treatment of complicated endocarditis improves outcome when compared to medical therapy alone, reducing 6-month mortality from 33 to 16% [6] and the composite endpoint of death/ embolic events/ recurrence of endocarditis from 28 to 3% [7]. Aortic valve paravalvular abscess formation and root destruction requires radical resection of infected tissue with subsequent reconstruction of the left ventricular outflow tract (LVOT) (modified Bentall procedure) [8]. Therefore, surgical treatment of complicated aortic valve endocarditis is considered challenging, bearing high operative (11–40% in-hospital) and late (60% in 5 years) mortality rates [9].

Various surgical techniques are used to treat complicated aortic valve endocarditis, depending on the surgical preference and with differing results: patch, prosthesis, homograft. Historically, cryopreserved homografts were considered as the gold standard for these patients [10–12]. Homografts offer low recurrence rates, acceptable valve-related morbidity and mortality, and their low transvalvular gradient is associated with improved left ventricular mass regression [13, 14]. Homografts also have disadvantages, including demanding surgical techniques, the need for reoperation due to calcification, limited availability and shelf life [8, 9, 15]. Nowadays, biological stentless valves are more often used in complicated aortic valve endocarditis [8, 9]. Using these prostheses, the surgical versatility of homografts is reached due to their comparable durability, shape and pliability [8]. In addition, stentless bioprostheses have advantages, such as a rather long shelf life and being readily available in various sizes, uniform in quality, technically easier to implant and furnished with anticalcification properties [8, 13, 14, 16–18].

Guidelines support the use of both homografts and stentless bioprostheses in aortic valve endocarditis with paravalvular abscess formation [2, 10, 19]. The choice of prosthesis depends on patient characteristics, technical considerations, and surgeon preferences [8, 14]. In this illustrated series of sixteen patients with aortic valve endocarditis and complicating paravalvular abscess formation, we show that the use of stentless bioprostheses provides a more standardized surgical procedure that consists of thorough debridement, root replacement with reimplantation of the coronary arteries, and treatment of accompanying pathologies.

## Methods

### Patients

In this case series we aimed at providing evidence for the standardized use of a stentless bioprostheses in complex aortic valve endocarditis. “Standardized use” refers to the use of one type of stentless bioprosthesis for a variety of anatomical problems complicating aortic valve endocarditis. Clinical data and high quality macroscopic pictures from sixteen patients with active aortic valve endocarditis and paravalvular abscess formation were collected between 2006 and 2015. In this time period, a total of 85 patients underwent aortic valve surgery for endocarditis in our center. Here, we report on those patients treated with stentless bioprostheses. Their endocarditis was not limited to the cusps but also involved the annulus with formation of large paravalvular abscesses at various anatomical locations. Consequently, complications arose, such as root disarrangement with loss of aortaventricular or aortomitral continuity, atrioventricular conduction disturbance, or infection of the septum or the right ventricle. Despite their poor clinical condition, these patients were deemed eligibile for surgical valve repair and LVOT reconstruction using stentless bioprostheses.

### Definitions

Infective endocarditis was diagnosed based on the modified Duke criteria [4] and expert opinion of a multidisciplinairy team. Prosthetic valve endocarditis was considered early if it occurred during the first year after valve replacement, otherwise it was considered late [2]. Causative micro-organisms were identified by culture and molecular testing on peripheral blood and tissue or prosthetic material collected during surgery [2]. Functional cardiac derangements as described by the guidelines were important indications for surgery [2, 19]. Macroscopically visible pathological findings considered an indication for the use of stentless bioprostheses were presence of destructive lesions, including annular abscess, paravalvular leak and cusp perforation. Re-thoracotomy was defined as reopening of the sternum after implantation of the bioprosthesis. Reoperation was defined as any surgical procedure involving the implanted bioprosthesis. Recurrence was used as a combined term for both relapse (repeat episodes of endocarditis caused by the same microorganism) and reinfection (infection caused by a different microorganism) [2].

### Prosthesis

**Fig. 1:**
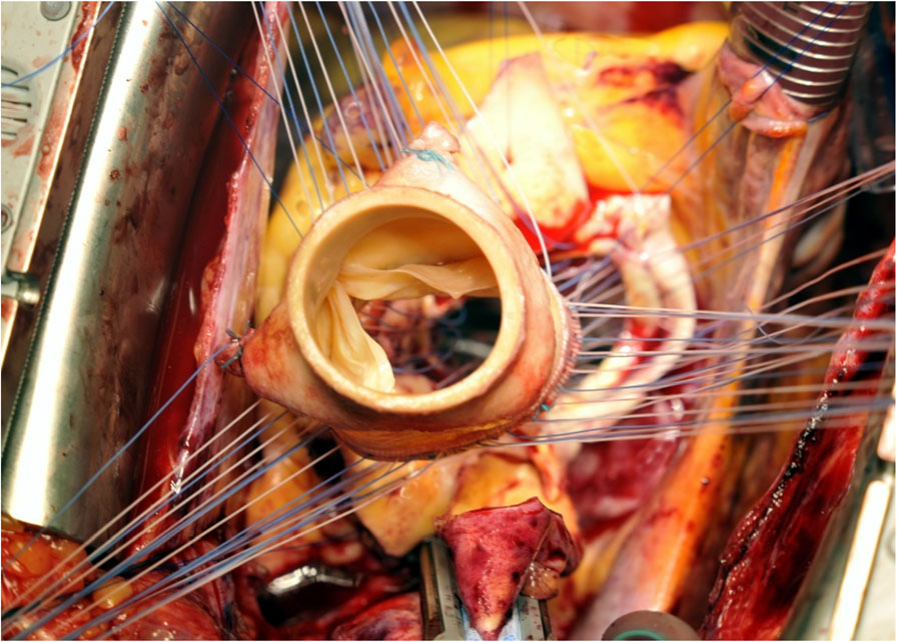
Stentless bioprosthesis

The Freestyle® bioprosthesis (Medtronic Inc., Minneapolis, MN, USA) is a stentless porcine aortic root prosthesis with ligated coronary arteries and a thin skirt over the porcine septal myocardium. The bioprosthesis is fixed with low pressure applied to the aortic wall, and zero-net pressure across the leaflets (Fig. 1). Pre-implantation, the bioprosthesis underwent an anticalcification treatment using alpha-amino-oleic acid. The device can be implanted by various techniques: subcoronary valve replacement, root inclusion, or complete aortic root replacement.

### Surgical technique

The standard surgical approach was a median (re)sternotomy with mild/moderate hypothermic (32–34 °C) cardiopulmonary bypass and cardioplegic cardiac arrest (retrograde blood cardioplegia). Cardiopulmonary bypass was performed using aortic cannulation and right atrial or bicaval cannulation for venous drainage.

**Fig. 2:**
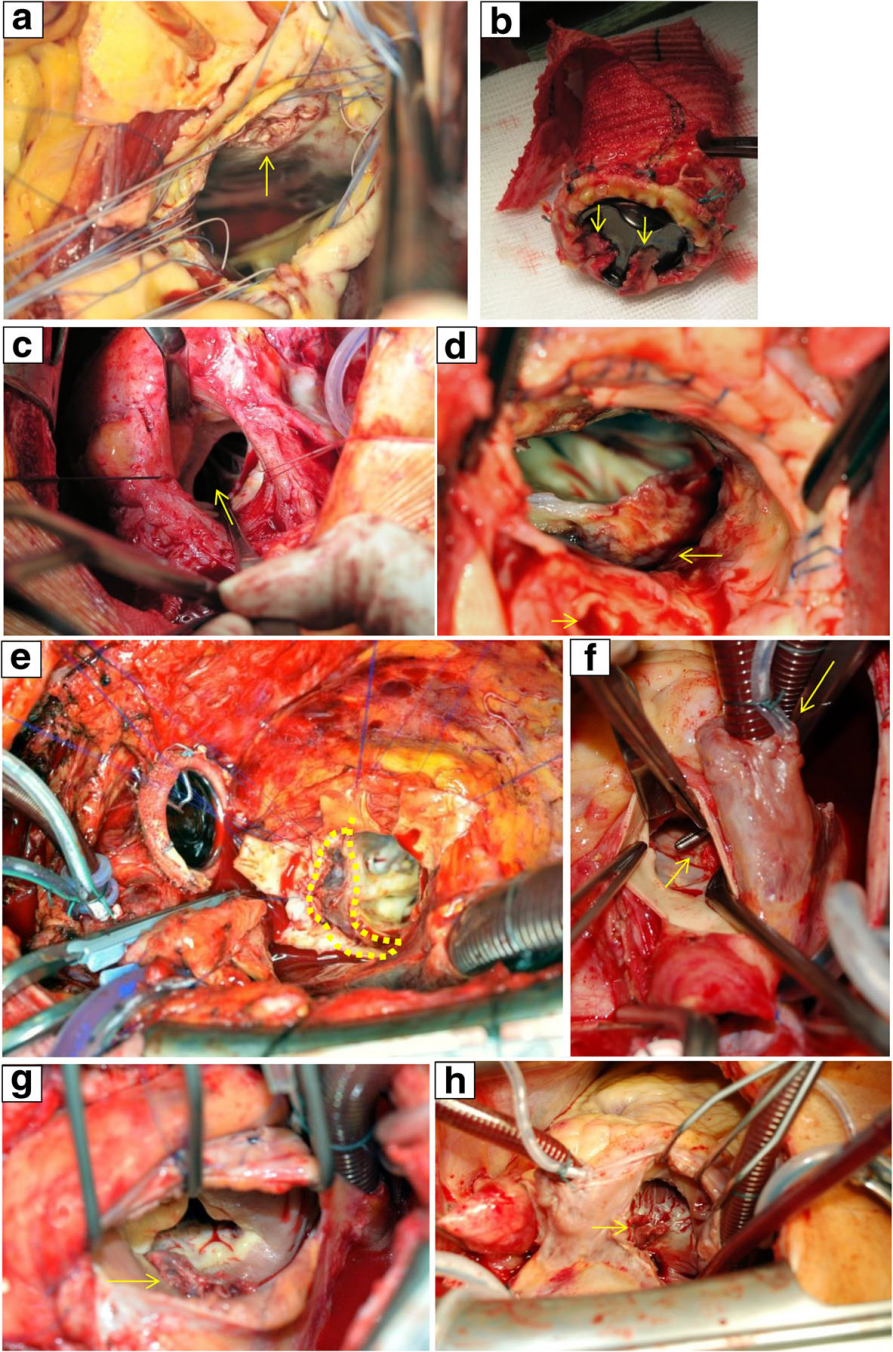
Aortic valve endocarditis with paravalvular abscess formation, surgical view: **a** view from aortic root, ventricular septal defect, **b** valved conduit with vegetations, **c** total aorto-ventricular dehiscence, with left ventricular outflow tract discontinuity, **d** abscess cavity (large arrow) with left main coronary visible (small arrow), **e** retro-aortal abscess cavity with aorto-mitral involvement and mitral annulus dehiscence, **f** aorto-atrial fistula, Gerbode-like defect, **g** atrial view, tricuspid valve annular abscess with torn septal leaflet and paravalvular leak, **h** tricuspid valve deformity with vegetational mass

The aorta was transected above the sinotubular junction. After the aortotomy exposure, the abscess regions were inspected (Fig. 2) and infected native cusps or prosthesis as well as any aortic aneurysms were removed with extensive tissue debridement. The aortic sinuses were resected with trumpet-shaped recesses of the coronary ostia. More specifically, a ventricular septum defect just under the membranous septum was identified in Fig. 2a. In this case a pericardial patch was used, which was distally sutured on the septum covering both the defect and the membranous septum, proximally attached at the level of the aortic annulus. Figure 2b and c depict chronic dehiscence of a mechanical prosthesis (implanted after a Bentall procedure) as a result of abcess formation at the annular level. Interestingly, the prosthesis was found floating above the annulus, only attached by the coronary arteries. Hence, the adhesions surrounding the annulus kept the prosthesis in place. Following resection of the infected prosthesis and clearance of the abcess, the stentless bioprosthesis was sutured on the annulus using a single-stitch technique. Given the chronic nature of disease in this case, the bioprosthesis was parachutted downwards towars the subannular plane to minimize traction of the chronically anchored anterior mitral leaflet (AML). In contrast, Fig. 2d and e illustrate acute subannular abcess formation. In this case, the AML was detached from the annulus while the prosthesis attachment site remained intact. In this case, due to the recent onset of infection, traction of the AML to the annulus plane and a neo-annulus were created after clearance of the abcess and other inflammatory tissue. Afterwards the stentless bioprosthesis was sutured to the annulus. Figure 2f to h depict Gerbode lesions with tricuspid valve involvement. Gerbode(−like) lesions encompass fistulas formed between the left ventricle(aorta) and the right side of the heart, appearing above or below the septal leaflet of the tricuspid valve. Repair of the sub-valvular fistula from the right side included temporary resection of the spetal leaflet of the tricupid valve, which was thereafter re-attached.

After debridement, restoration and sizing of the aortic annulus the proximal anastomosis was performed using 20–25 interrupted sutures of Ticron 3–0 in a single plane. If required, the coronary ostia were mobilised using diathermy. After completion of the proximal suture line, the patient’s coronary ostia were reimplanted end-to-side to the corresponding sinus of Valsalva of the prosthesis using a continuous 5–0 polyproylene suture. Finally, the bioprosthesis was anastomosed with the aorta using continuous 4–0 polyproylene. If further resection of the ascending aorta was required, a vascular tube graft was interposed.

### Ethical considerations

The institutional medical ethical review board of the University Medical Center Groningen approved the use of retrospective patient data for our study and waived informed consent (METc2015/033; February 2015).

## Results

### Patient characteristics

This series consecutively included 14 males and 2 females with a median age of 63 years (Tables 1 and 2). All patients had an urgent indication for cardiothoracic surgery with implantation of a stentless bioprosthesis as root replacement due to uncontrolled infection and abscess formation (evidence class I and level B [2]). Median New York Heart Association score was III, and median logarithmic EuroSCORE I score was 40.7. Median follow-up for survivors was 4.6 years. All survivors were followed for at least 2 years, 36% were followed for 5 years, and 9% for 10 or more years. In 4 patients (25%) the endocarditis involved native aortic valves, with 2 identified bicuspid valves. In 12 patients (58%) the endocarditis involved prosthetic aortic valves: in 7 patients the aortic valve was replaced once and in 1 patient twice before, in 5 patients a Bentall procedure had been performed. Of the patients with prosthetic valves, 2 patients had early (3–4 months after surgery) and 10 patients late endocarditis (1–29 years after surgery). In-hospital and 30 day mortality were 18.8% and 12.5%, respectively; 2-year recurrence rate was 14%.

**Table 1:** Patient characteristics (*n* = 16

Characteristic	Value
Age: median [range] (years)	63 [31–77]
Gender: male; female, n (%)	14 (87.5); 2 (12.5)
Reoperation / PVE (%)	75
Follow-up survivors: median [range] (years)	4.6 [2.3–11.7]
NYHA score: median [range]	III [II-IV]
Logarithmic EuroSCORE I: median [range]	40.7 [12.8–68.3]
Microbiology	
*PVE*	
*• Staphylococcus* spp.: 5 CoNS, 1 *S. aureus*	*n* = 6 (50%)
*• Streptococcus* spp.: 1 viridans group, 1 *S. bovis*, 1 *S. agalactiae*	*n* = 3 (25%)
*• Enterococcus* spp.: 2 *E. faecalis*	*n* = 2 (17%)
*•* no micro-organism identified	*n* = 1 (8%)
*NVE*	
*• Staphylococcus* spp.: *S. aureus*	*n* = 1 (25%)
*• Streptococcus* spp.: 2 viridans group	*n* = 2 (50%)
*• Enterococcus* spp.: *E. faecalis*	*n* = 1 (25%)
Outcome	Value
Cardiopulmonary bypass perfusion time: median [range] (minutes)	358 [186–731]
Aortic cross-clamping time: median [range] (minutes)	266 [107–389]
Intensive care unit stay: median [range] (days)	1.5 [1–21]
Hospital stay: median [range] (days)	55 [29–90]
In-hospital mortality: n (%)	3 (18.8)
30 day mortality: n (%)	2 (12.5)

*CoNS* coagulase negative staphylococci, *COPD* chronic obstructive pulmonary disease, *e.c.i.* e cause ignota, *NVE* native valve endocarditis, *NYHA* New York Heart Association, *PVE* prosthetic valve endocarditis, *SD* standard deviation

**Table 2:** Characteristics of included patients

#	Age (yr)	sex	Previous surgery	Micro-organism	Indication for surgery	Euro SCORE	Remarks during stentless bioprosthesis implantation	Outcome			
								rethoracotomy	re-IE	permanent dialysis	PPM
1	66	M	2 yr. bio	*Streptococcus sanguinis*	aortic root abscess	38.92	pericard patch to support MV, 1 RBC	Recovery initially, but death 7.5 months post surgery			
								–	+	–	+
2	70	M	1 yr. bio	*Staphylococcus epidermidis*	aortic root abscess, mycotic aneurysm, loose prosthesis, septic emboli, AV block	65.87	aorta annulus support with pledges and transseptal stiches, CABG, 5 RBC	In-hospital death 40 days post surgery			
								–	–	–	+
3	71	M	1 yr. bio	*Streptococcus agalactiae*	aortic root abscess with Gerbode defect, AV block	47.06	pericard patch reconstruction aorta annulus, atriotomy, TVP and Devega plasty, 14 RBC	Recovery > 6 years post surgery			
								–	–	–	+
4	31	M	–	*Streptococcus mitis*	totally destructed LVOT with Gerbode defect, AV block	42.52	pericard patch reconstruction aorta annulus, TVP, Devega plasty, 0 RBC	Recovery > 4 years post surgery			
								–	–	–	+
5	71	M	29 yr. mech	*Enterococcus faecalis*	aortic root abscess, septic emboli	47.06	3 RBC	Recovery > 3 years post surgery			
								–	–	–	–
6	36	M	2 yr. mech	not identified	aortic root abscess, septic emboli	28.55	0 RBC	Recovery > 4 years post surgery			
								–	–	–	–
7	64	M	–	*Staphylococcus aureus*	aortic root abscess, multiple septic emboli, cardiac decompensation	23.42	aorta annulus support with pledges, 2 RBC	Recovery > 2 year (20 months) post surgery			
								–	–	–	+
8	72	M	3mo bio	*Staphylococcus epidermidis*	loose prosthesis, cardiac decompensation	64.48	closure of destructed coronary ostia, CABG, 0 RBC	In-hospital death 14 days post surgery			
								–	–	–	–
9	45	M	12 yr. mech	*Staphylococcus aureus*	aortic root abscess, mycotic aneurysm	28.55	multiple vegetations AV, pericard patch reconstruction aorta annulus, 0 RBC	Recovery initially, but death 13 months post surgery			
								–	+	–	+
10	60	F	4mo bio	*Staphylcoccus epidermidis*	progressive aortic root abscess with Gerbode defect, septic emboli, blood cultures persistantly positive, AV-block	37.28	removal of vegetation from right atrium with affected AML and PPM implantation, 4 RBC	Recovery > 2 years post surgery			
								–	–	–	+
11	55	M	–	*Enterococcus faecalis*	aortic root abscess, mycotic aneurysm, conduction disturbance	26.62	pericard patch reconstruction aorta annulus and AML, 1 RBC	Recovery > 4 years post surgery			
								–	–	–	–
12	42	M	–	*Streptococcus mutans*	mycotic aneurysm, large vegetation	12.79	MVP, 0 RBC	Recovery > 5 years post surgery			
								–	–	–	–
13	75	F	1 yr. bio	*Staphylococcus epidermidis*	aortic wall thickening, septic emboli, AV block	61.76	mobilization of tightly adhered coronary ostia, 2 RBC	Recovery > 8 years post surgery			
								–	–	–	+
14	77	M	2 yr. bio	*Enterococcus faecalis*	septal mycotic aneurysm with fistula and threatened anatomy	52.33	urgent surgery with two times reanimation setting and persistant instability for which sternum left open, 0 RBC	In-hospital death directly post surgery			
								–	–	–	–
15	62	M	1 yr. mech	coagulase negative Staphylococci	aortic root abscess, progressive mycotic aneurysm, aortoventricular dehiscence	68.31	4 RBC	Recovery > 11 years post surgery			
								–	–	–	–
16	60	M	7 yr. mech	*Streptococcus bovis*	aortic root abscess, mycotic aneurysm, aortoventricular dehiscence, cardiac decompensation	60.7	drainage of 1 L pleural effusion at both sides, 0 RBC	Recovery > 5 years post surgery			
								–	–	–	–

# patient number, *AML* anterior mitral leaflet, *AV* aortic valve, *AV block* atrio-ventricular block, *bio* biological prosthetic valve inplanted, *CABG* coronary artery bypass grafting, *EuroSCORE* logarithimic I, *F* female, *LVOT* left ventricular outflow tract, *M* male, *mech* mechanical prosthetic valve inplanted, *mo* months, *MV* mitral valve, *PPM* placement of permanent pacemaker, *RBC* number of bags with red blood cells given during surgery, *re-IE* recurrence of endocarditis, *rethoracotomy* for bleeding or tamponade, *TVP* tricuspid valve plasty, *yr.* years

### Infectious cardiac anatomical compliations eligible for stentless bioprostheses repair

**Fig. 3:**
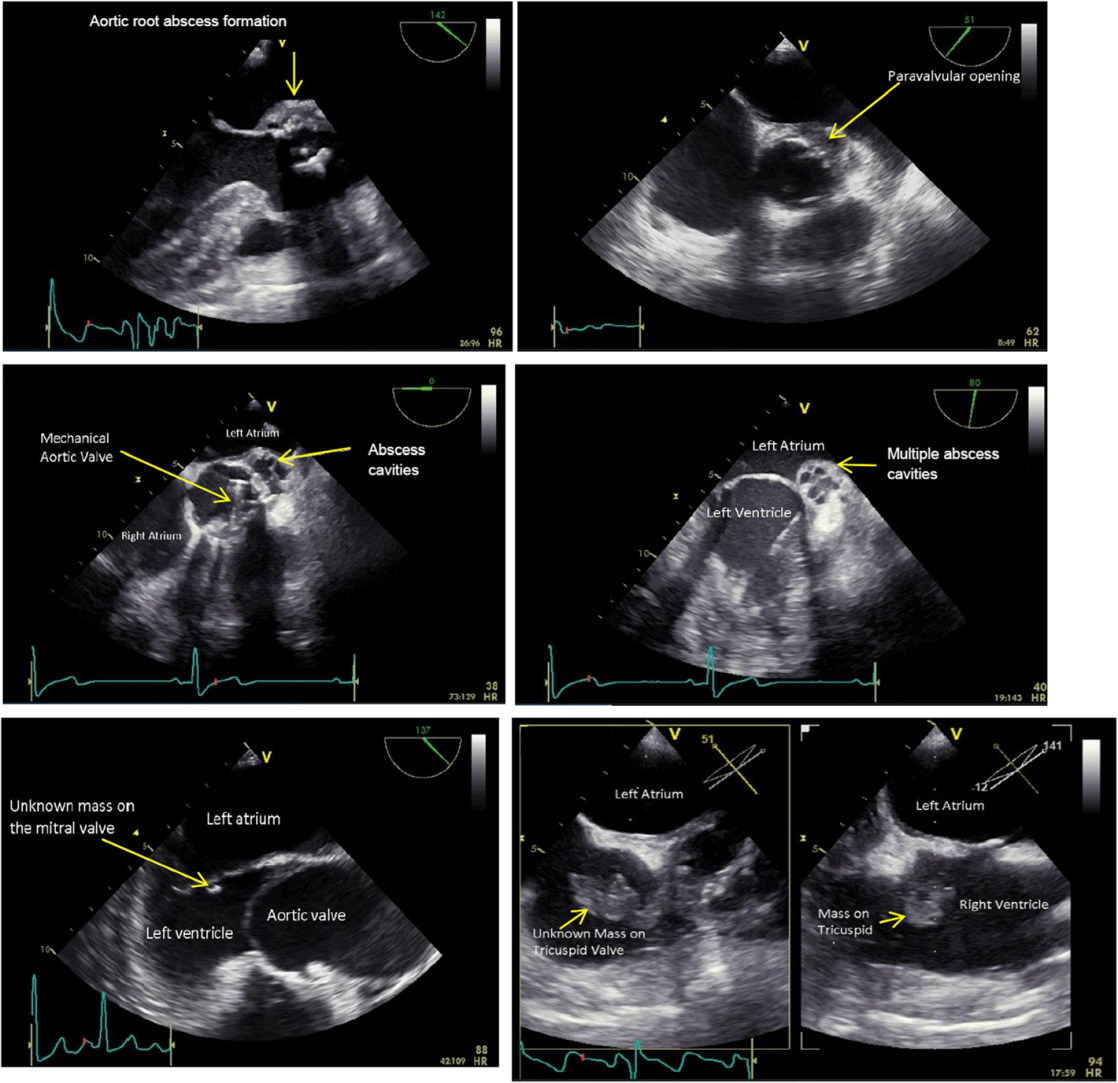
Aortic valve endocarditis with paravalvular abscess formation, transesophageal echocardiographic view

**Fig. 4:**
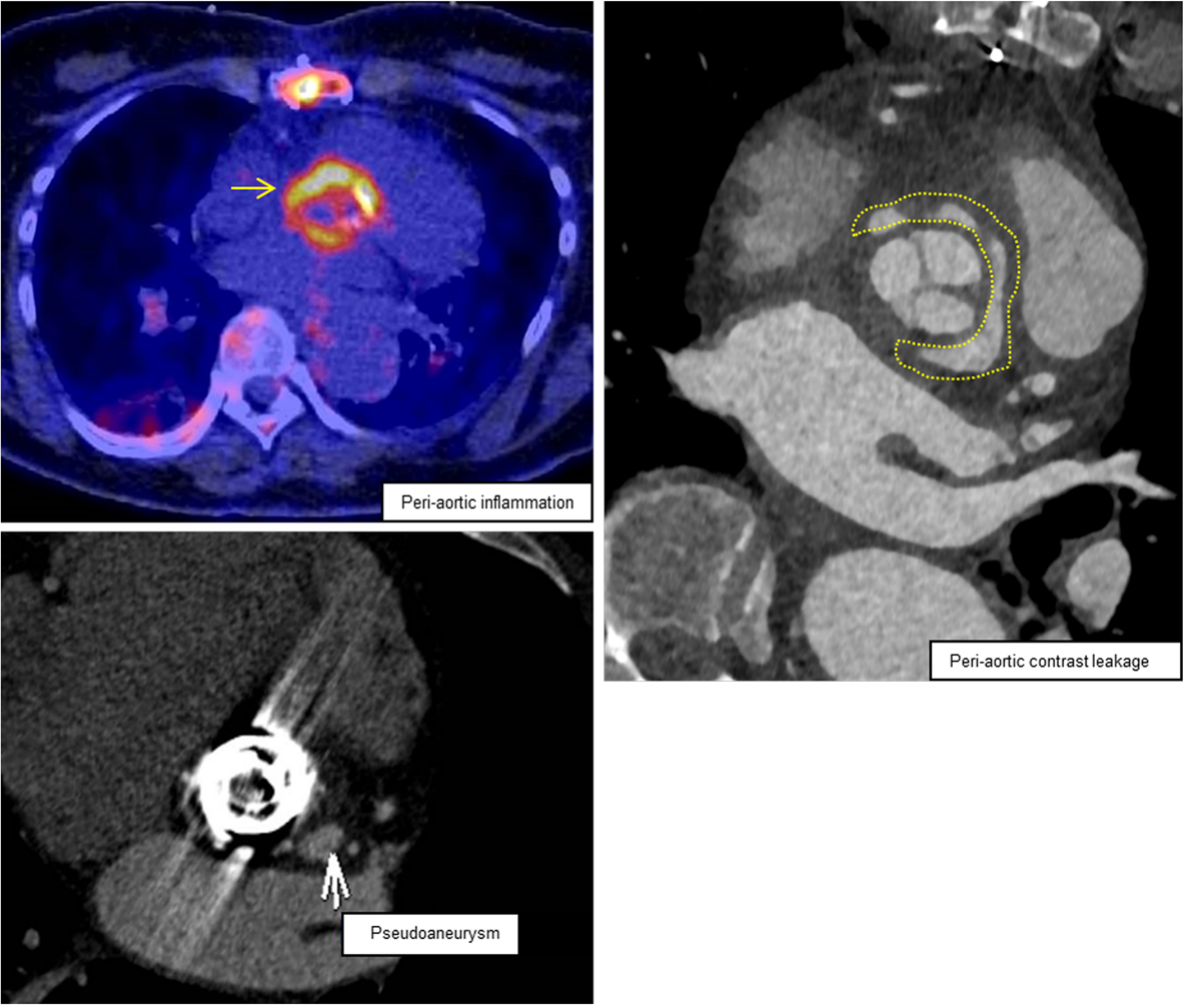
Aortic valve endocarditis with paravalvular abscess formation, nuclear/radiological view with ^18^F-fluorodeoxyglucose positron emission tomography/computed tomography

**Fig. 5:**
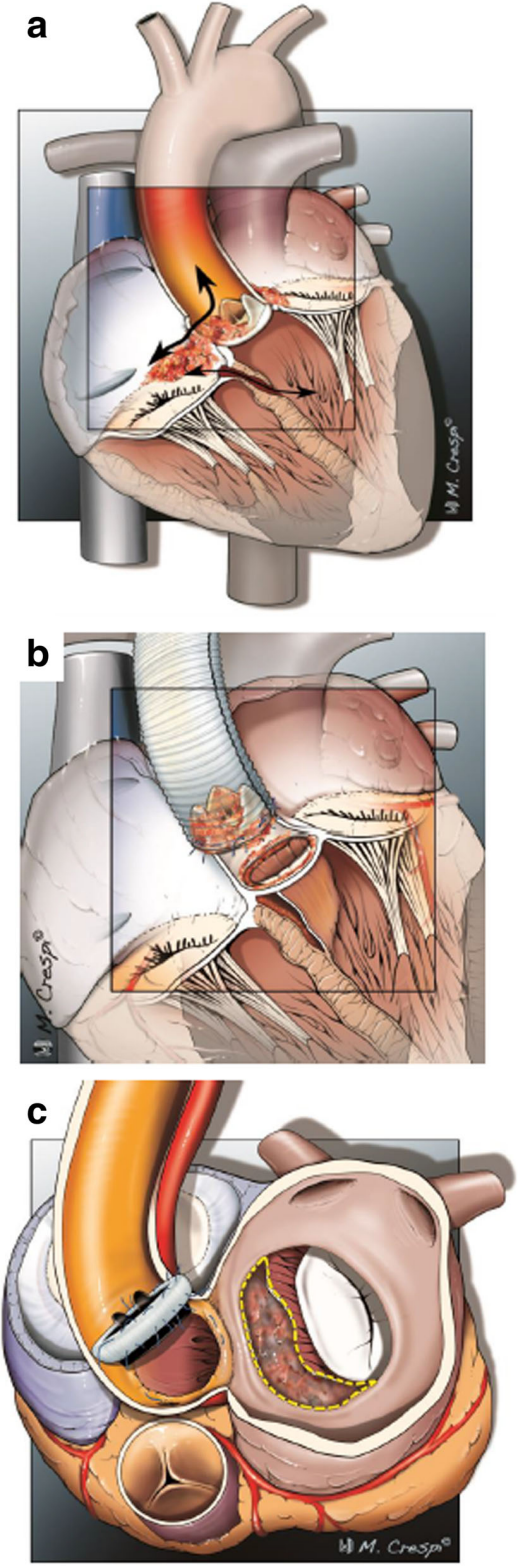
Aortic valve endocarditis with paravalvular abscess formation, illustrations: **a** coronal view on the heart showing a ventricular septum defect, Gerbode defect (communication between the left ventricle and the right atrium), Gerbode-like defect (communication between the aorta and the right atrium) and tricuspid valve deformity; **b** coronal view on the proximal heart showing total aorto-ventricular dehiscence; **c** horizontal view on the proximal heart showing a retro-aortal abscess cavity with aorto-mitral involvement and mitral annulus dehiscence

Several situations of active aortic valve endocarditis with paravalvular abscess formation and accompanying pathologies were deemed eligible for valve repair and LVOT reconstruction with a stentless bioprosthesis (Figs. 2, 3, 4 and 5).

#### Aortoventricular dehiscence

Seven patients with a prosthetic valve presented with aortoventricular dehiscence. Pathogens included coagulase-negative staphylococci, *Staphylococcus aureus*, *Streptococcus* bovis, *Enterococcus faecalis*. 29% (2/7) of these patients also had extention of infection towards their mitral valve.

#### Septum derangements

Seven patients presented with infectious derangements of their septum, including vegetations and perforations. Four of these patients had a prosthetic valve. Pathogens included *Staphylococcus epidermidis*, *Staphylococcus aureus*, *Streptococcus agalactiae*, *Streptococcus mitis*, and *Enterococcus faecalis*. 71% (5/7) of these patients had a permanent pacemaker (PPM) implanted and 43% (3/7) had extention of infection towards the right side of the heart through a Gerbode(−like) defect.

#### Total atrioventricular conduction block

Six patients presented with a total or third degree atrioventricular conduction block. Five of these patients had a prosthetic valve. Pathogens included *Staphylococcus epidermidis*, *Streptococcus sanguinis, Streptococcus agalactiae*, and *Streptococcus mitis*. All these patients had a PPM implanted, 50% had extention of infection towards their right ventricle through a Gerbode(−like) defect, and 50% had extention of infection towards their mitral valve.

#### Gerbode defect (with tricuspid valve involvement)

Three patients presented with a Gerbode(−like) defect, a left ventricular (aorta) to right atrial shunt [20], causing an infection of their tricuspid valve due to local spread. Two of these patients had a prosthetic valve. Pathogens included *Staphylococcus epidermidis*, *Streptococcus agalactiae* and *Streptococcus mitis*. All patients had a PPM implanted, and needed a tricuspid valve plasty.

#### Mitral valve involvement (with aortomitral dehiscence)

Seven patients presented with extension of infection towards their mitral valve. Five of these patients had a prosthetic valve. Pathogens included *Staphylococcus epidermidis*, *Streptococcus sanguinis*, *Streptococcus mutans*, and *Enterococcus faecalis*. 57% (4/7) of these patients had septic emboli.

### Extracardiac complications due to endocarditis

Infective endocarditis is a cardiac disease with extracardiac complications due to hematogenous and embolic spread. In our series, the three most common complications were: mycotic aneurysm (*n* = 3), cerebral emboli (*n* = 2), and vertebral osteomyelitis (*n* = 2).

### Patient survival

**Fig. 6:**
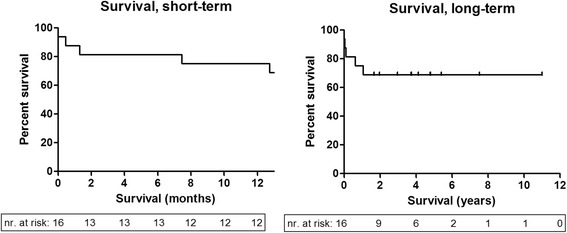
Kaplan-Meier curves. The short-term curve depicts the survival of included patients over 12 months post surgery and the long-term curve depicts the survival of included patients during the total follow-up time (maximum 11 years)

Figure 6 shows the survival of included patients for 11 years. Five patients died during this period (Table 2), due to: end-stage heart failure 227 days post-surgery; recurrent respiratory insufficiency resulting from sputum retention, encephalopathy and extended postoperative wound infection 40 days post-surgery; active intra-cerebral bleeding without therapeutic options 14 days post-surgery; re-infection of the prosthesis with cerebral embolization, mediastinitis and kidney failure 388 days post-surgery; severe hemodynamic instability immediately post-surgery.

## Discussion

We have described and illustrated a series of patients with aortic valve endocarditis, paravalvular abscess formation and accompanying pathologies. All patients underwent cardiothoracic surgery with thorough debridement and restoration of cardiac anatomy using stentless bioprostheses. Patients with native and several types of prosthetic valves were included. Pathogens varied, including staphylococci (*n* = 7), streptococci (*n* = 5) and enterococci (*n* = 3). Predicted mortality was high (median logarithmic EuroSCORE I of 40.7 [range 12.8–68.3]) but actual mortality was relatively low (in-hospital 18.8% [3/16] and 30-day 12.5% [2/16]), showing that the stentless bioprostheses can be successfully used in a variety of surgically challenging situations and allows for a standardized approach. Figures 2, 3 and 4 show the cases of aortic valve endocarditis with various paravalvular abscesses from a surgical (Fig. 2), echocardiographic (Fig. 3) and nuclear/radiological (Fig. 4) view.

Due to its design, it is possible to use the stentless bioprostheses for subcoronary valve replacement, for inclusion of the root, or full root replacement [13, 16]. Using the prostheses for a full root replacement, enables exclusion of abscess cavities and the rebuilding of the LVOT. Furthermore, it maintains root geometry and the integrity of the “leaflet, sinus and root” as a functional entity, both increasing durability of the bioprostheses [8, 21]. Implantation with the single suture technique is believed to allow placement of the stentless bioprosthesis as full root replacement without narrowing of the LVOT nor obstruction by any rigid structures such as pledgets [21]. Using the stentless bioprothesis as a full root replacement in complex endocarditis was previously reported in 5 patients [22] and now supported with our data of 16 patients with various well described paravalvular abscesses.

Survival rates for the use of stentless bioprostheses when active native or prosthetic aortic valve endocarditis is complicated by extensive destruction of the LVOT have been reported as 81–89%, 76–83%, 62–70%, and 54% at 30 days, 1 year, 5 years, and 10 years, respectively [8, 13, 14, 16, 17]. Although early mortality remains considerably high in the group presented, studies show that stentless bioprostheses yield clinical outcomes, postoperative echocardiographic data, long-term recurrence and survival rates comparable to those of cryopreserved homografts [8, 9, 13, 14, 16, 17]. Indeed, the recurrence rates of homografts (3.8–6.8%) and stentless valves (3.7–8.6%) are similar and lower than that of standard prostheses (33%) [13]. However, as compared with standard aortic valve replacement, the need for re-implantation of coronary arteries conveys an increased risk of atrioventricular conduction block. Also, the use of bioprosthesis conveys an increased risk of reoperation in juvenile patients. Even though stentless and stented valves show equal performance with regard to clinical parameters and valve-related mortality, stentless valves have more favorable hemodynamic and biomechanical characteristics and significantly higher long-term survival rates (78% versus 66% in 8-years) [8, 14, 16]. Compared to homografts, progression of valve dysfunction (37% versus 86%, *p* < 0.01) [23] and need for reoperations are lower for stentless bioprostheses [14, 18, 23]. Furthermore, implantation is less challenging and demanding for stentless bioprostheses and reoperation of a calcified prosthesis may be easier as compared to homografts [9].

A limitation of this study is its retrospective nature. Furthermore, we did not directly compare the Freestyle® bioprosthesis with other stentless bioprostheses, nor with homografts. The described patient group had been previously treated with homografts, but we did not consider it useful to compare results from 10 years ago with recent results. Prospective studies should examine durability and long-term valve-related complication free survival of patients treated with various models of stentless bioprostheses. Experience with reoperation for replacing a bioroot also needs further examination [21].

## Conclusion

Aortic valve endocarditis with paravalvular abscess formation remains a therapeutic challenge for which stentless bioprosthesis is a credible surgical option. This prosthesis allows a radical and uniform approach with a good surgical overview and use of limited prosthetic material. It enables successful treatment of complex aortic valve endocarditis with complete debridement, elimination of shunts and anatomical deviations, reconstruction of the LVOT and aortomitral continuity. Stentless bioprostheses yield comparable clinical outcomes as the historical gold standard – the homograft – and are readily available. Of note, use of one type of prosthesis reduces logistical issues and purchasing costs.

## Abbreviations

AML: Anterior mitral leaflet
LVOT: Left ventricular outflow tract
PPM: Permanent pacemaker
